# Sliding Mode Fault Tolerant Control for Unmanned Aerial Vehicle with Sensor and Actuator Faults †

**DOI:** 10.3390/s19030643

**Published:** 2019-02-03

**Authors:** Juan Tan, Yonghua Fan, Pengpeng Yan, Chun Wang, Hao Feng

**Affiliations:** 1 School of Astronautics, Northwestern Polytechnical University, Xi’an 710072, China; tanjuan@mail.nwpu.edu.cn (J.T.); ypp@mail.nwpu.edu.cn (P.Y.); wang_chun@mail.nwpu.edu.cn (C.W.); 2 Shanghai Aerospace Control Technology Institute, Shanghai 201109, China; 13152062031@mail.nwpu.edu.cn

**Keywords:** unmanned aerial vehicle (UAV), sensor faults, actuator faults, fault-tolerant control, sliding mode control (SMC)

## Abstract

The unmanned aerial vehicle (UAV) has been developing rapidly recently, and the safety and the reliability of the UAV are significant to the mission execution and the life of UAV. Sensor and actuator failures of a UAV are one of the most common malfunctions, threating the safety and life of the UAV. Fault-tolerant control technology is an effective method to improve the reliability and safety of UAV, which also contributes to vehicle health management (VHM). This paper deals with the sliding mode fault-tolerant control of the UAV, considering the failures of sensor and actuator. Firstly, a terminal sliding surface is designed to ensure the state of the system on the sliding mode surface throughout the control process based on the simplified coupling dynamic model. Then, the sliding mode control (SMC) method combined with the RBF neural network algorithm is used to design the parameters of the sliding mode controller, and with this, the efficiency of the design process is improved and system chattering is minimized. Finally, the Simulink simulations are carried out using a fault tolerance controller under the conditions where accelerometer sensor, gyroscope sensor or actuator failures is assumed. The results show that the proposed control strategy is quite an effective method for the control of UAVs with accelerometer sensor, gyroscope sensor or actuator failures.

## 1. Introduction

Currently, with the exploration, development and utilization of aerospace and aircraft fields increasing significantly, the low-cost unmanned aerial vehicle (UAV) has attracted more and more attention among many of the world’s powers [1,2,3,4]. The UAV flies mostly in the troposphere near the earth, which implies complex weather conditions such that the complex structures, such as the sensor and actuator of the UAV system, are more likely to lead to accident. Therefore, while using UAVs to carry out various missions has attracted more and more attention and also gained satisfying results [5], the method of how to reduce the accident risk of UAV is vital to extend the application of UAV further. 

Sensor and actuator, key components to sense the flight state of UAV and control UAV, respectively, are some of the components most likely to fail due to their complex structure, hostile working environment, various unknown disturbances, and uncertain factors [6,7]. Thus, the fault tolerance control method of UAV is studied to implement effective control for possible faults of sensors and actuators [8,9,10], which is a necessary means to improving the reliability and safety of UAV, reduce potential safety risks, and prevent catastrophic accidents of the system, making the vehicle health management more convenient [11,12]. Research on fault detection and isolation (FDI) [13,14,15] and fault tolerant control (FTC) [16,17], from both theoretical and practical perspectives, has received more attention in recent years. Advanced fault-tolerant control (FTC) systems are designed to help pilots overcome abnormal situations that previously might have resulted in catastrophic events [18]. 

The sensor of UAV mainly includes an accelerometer and gyroscope. When it fails, a series of wrong feedbacks will be introduced, causing a lot of trouble to the accuracy of control commands [19]. The actuator failure deteriorates the control performance and affects the stability and security of UAV, even leading to catastrophic accidents. In the current papers, efforts have been made for fault-tolerant control schemes for actuator or sensor faults. Wang [20] designed an adaptive sliding mode passive fault-tolerant controller with finite time convergence for the actuator’s failure of the elastic hypersonic vehicle model. For actuator stuck faults, the authors of References [21,22,23] provide direct adaptive fault-tolerant control approaches. Gao [24] investigated the robust fault tolerant tracking problem for a linearized hypersonic vehicle model with bounded external disturbance and sensor faults. M. Chadli [18] designed the FTC for a Vertical Takeoff and Landing (VTOL) aircraft subject to external disturbances and actuator faults based on a faulty T-S uncertain disturbed model. The research on accommodating actuator and sensor failures and maintaining acceptable system performance continues to attract considerable attention from control engineers and motivated us to do this study.

As a typical robust control method, sliding mode control (SMC) scheme is regarded as an effective method to cope with external disturbance and parametric uncertainties [25,26,27]. Recently, the SMC method has been widely applied for fault tolerant control of aircraft systems, spacecrafts, and so on [28,29]. Zhang [30] proposed a method combining feedback linearization (FBL) and sliding mode control for reusable launch vehicle (RLV) to minimize the impact of control effector failures or damage. In Reference [31], a fault-tolerant sliding mode controller was presented for an aircraft system, which requires the message of the effectiveness factor, while it may be difficult and expensive to obtain the actuator faults online. Nguyen and Hong [32] designed an adaptive sliding mode Fault-Tolerant Control that can handle system uncertainties and actuator faults for quadcopter UAVs on the basis of normal adaptive sliding mode control and using RBF for fault identification and reconstruction. However, the method of Nguyen and Hong is based on the traditional linear sliding surface. Zeghlache [4] designed a fault tolerant control using Radial-Based Function Neural Network (RBFNN) fuzzy sliding mode for coaxial octorotor UAV, in which RBFNN is used to approximate the unknown part of the octorotor helicopter dynamic equation. By his approach, the approximation error, disturbance and the effects of faults can be compensated by the sliding mode control. The aforementioned references could achieve the desired performance through SMC methodology, taking sensor and actuator faults into consideration. However, they all adopt a linear sliding surface which results in the system states and the errors not being able to converge to an equilibrium point asymptotically in finite time. In other words, it means that finite-time convergence is not ensured. Motivated by the above discussions, the RBF combining the terminal sliding mode control were proposed to design the fault tolerant controller with actuator and sensor failures to achieve finite-time convergence. What is more, with the assistance of RBF, the efficiency of the design process is improved and the system chattering is minimized. However, to the best of the authors’ knowledge, there are few researches concentrating on using RBF to adjust parameters for the sliding mode controller.

The objective of this study is to exploit the effectiveness of the FTC law for the overload tracking problem of a UAV system such that the closed loop system can maintain stability and performances for the actuator and sensor fault case. The main contribution of this paper is that it proposes a robust sliding mode fault tolerant control approach in the presence of actuator and sensor faults. The proposed method has considered RBF, which is used to design the parameters of the sliding mode controller and to attenuate the chattering. The rest of this paper is organized as follows. In Section 2, the simplified dynamic model of UVA is established. In Section 3, the terminal Sliding Mode Controller is designed and the stability of Sliding Mode is demonstrated, then the RBF Neural Network algorithm is used to design the parameters of the sliding mode controller. Simulation results are discussed in Section 4, and the conclusions are presented in Section 5.

## 2. The Simplified Dynamics Model of UAV

A simplified dynamic model of UVA is established in the body coordinate system with the consideration of coupling effects among pitch, yaw and roll channels.

(1) $$\left\{ \begin{array}{c} \dot{\alpha }=\omega_{z}-\beta \cdot \omega_{x}-a_{34}\alpha -a_{35}\delta_{z}\\ \dot{\beta }=\omega_{y}+\alpha \cdot \omega_{x}-b_{34}\alpha -b_{37}\delta_{y}\\ \dot{\omega_{x}}=-b_{11}\omega_{x}-b_{18}\delta_{x}-b_{14}\beta -\left(b_{12}\omega_{y}+b_{17}\delta_{y})+(J_{y}-J_{z})/J_{x}\cdot \omega_{y}\omega_{z}\right)\\ \dot{\omega_{y}}=-b_{24}\beta -b_{22}\omega_{y}-b_{27}\delta_{y}+\left(b_{21}\omega_{x}-b_{28}\delta_{x})+(J_{z}-J_{x})/J_{y}\cdot \omega_{z}\omega_{x}\right)\\ \dot{\omega_{z}}=-a_{24}\alpha -a_{22}\omega_{z}-b_{25}\delta_{z}+(J_{x}-J_{y})/J_{z}\cdot \omega_{x}\omega_{y} \end{array}\right.$$

where $\delta_{z},\delta_{y}\;\mathrm{and}\;\delta_{x}$ are the equivalent angle of pitch, yaw and roll rudder respectively and they are also the virtual controls in this paper. ${\dot{\omega }}_{z},$${\dot{\omega }}_{y}$ and ${\dot{\omega }}_{x}$ are pitch, yaw and roll angular velocity respectively. $a_{22},a_{24},a_{25},a_{34},a_{35},b_{22},b_{24},b_{27},b_{34},b_{37}b_{11},b_{17},b_{18},b_{14},b_{21},b_{28},\;\mathrm{and}\;b_{12}$ are the dynamic coefficients of UAV [33]. $J_{x},J_{y}\;\mathrm{and}\;J_{z}$ are the three channel moments of inertia. The coupling among the pitch, yaw and roll channels cannot be neglected by bank-to-turn (BTT) UAV, which can be listed as follows in Table 1.

## 3. Methods

### 3.1. The Design of Fault-Tolerant Control

The purpose of design of the terminal sliding mode fault-tolerant controller is to improve the robustness of system and to track the command signals precisely with sensor and actuator faults and coupling effects. To realize the efficient control of the simplified coupling model, coupling is often set as the disturbance term during the UAV controller design. The pitch, yaw and roll channels are separately designed, and then the simulation is carried out using a triple-channel control algorithm. The dynamic models of the three channels are as follows:

Pitch channel

$$\{\begin{array}{c} \dot{\alpha }=\omega_{z}-a_{34}\alpha -a_{35}\delta_{z}\\ \dot{\omega_{z}}=-a_{24}\alpha -a_{22}\omega_{z}-b_{25}\delta_{z}+(J_{x}-J_{y})/J_{z}\cdot \omega_{x}\omega_{y}\\ n_{y1}=a_{234}\alpha \cdot V/g \end{array}$$

Yaw channel

$$\{\begin{array}{c} \dot{\beta }=\omega_{y}+\alpha \cdot \omega_{x}-b_{34}\alpha -b_{37}\delta_{y}\\ \dot{\omega_{y}}=-b_{24}\beta -b_{22}\omega_{y}-b_{27}\delta_{y}+\left(b_{21}\omega_{x}-b_{28}\delta_{x})+(J_{z}-J_{x})/J_{y}\cdot \omega_{z}\omega_{x}\right)\\ n_{z1}=-b_{34}\beta \cdot V/g \end{array}$$

Roll channel

$$\{\begin{array}{c} \dot{\omega_{x}}=-b_{11}\omega_{x}-b_{18}\delta_{x}-b_{14}\beta -\left(b_{12}\omega_{y}+b_{17}\delta_{y})+(J_{y}-J_{z})/J_{x}\cdot \omega_{y}\omega_{z}\right)\\ \dot{\gamma }=\omega_{x}\\ \gamma =\int \dot{\gamma }\mathrm{dt} \end{array}$$

Due to the similarity among pitch, yaw and roll channels, the pitch channel will be derived only here.

#### 3.1.1. Selection of Sliding Surface

An appropriate sliding hyperplane can ensure the stability and dynamic quality of the sliding mode motion. The terminal adaptive sliding surface improved from the traditional integral sliding surface is designed to describe the error of state variables:

(2) $$s_{1}=-c_{1}(n_{y0}-n_{\mathrm{yc}})-\int_{0}^{t}c_{2}(n_{y0}-n_{\mathrm{yc}}{)}^{\frac{p}{q}}\mathrm{dt}-c_{3}(\omega_{z0}-\omega_{\mathrm{zc}})$$

where $c_{1},\;c_{2},\;\mathrm{and}\;c_{3}$ are the design parameters less than 0 and directly influence the time that the error converges to zero. $c_{1}$ is designed by pole assignment. $c_{2}\;\mathrm{and}\;c_{3}$ are chosen and adjusted according to the dynamic characteristics of the model using numerical simulation. When p/q is less than 1 and greater than 1/2, there is no singular problem. In this paper, 5 and 7 are chosen for p and q respectively.

#### 3.1.2. The Design of Control Law

The design of control law of the variable structure is indispensable in achieving the asymptotic stability of the sliding mode motion and satisfactory dynamic quality of system. This paper adopts a hyperbolic tangent function instead of a sign function to eliminate the chattering of the variable structure control. Then, the continuous reaching law is:

(3) $${\dot{s}}_{1}=-\rho_{1}\tanh\left(\mu^{-1}s_{1}\right)-k_{1}s_{1}$$

where μ is a small number, while $\rho_{1}$ and $k_{1}$ are the design parameters of the controller which are positive, and $k_{1}$ is obtained using the RBF neural network as shown below.

Differentiating the sliding surface of Equation (2)

(4) $${\dot{s}}_{1}=-c_{1}\left({\dot{n}}_{y0}-{\dot{n}}_{\mathrm{yc}}\right)-c_{2}{\left(n_{y0}-n_{\mathrm{yc}}\right)}^{\frac{5}{7}}-c_{3}\left({\dot{\omega }}_{z0}-{\dot{\omega }}_{\mathrm{zc}}\right)$$

When input commands are standard, namely, ${\dot{\omega }}_{z0}=0$, ${\dot{n}}_{\mathrm{yc}}=0$, substitute Equation (1) into Equation (4):

(5) $${\dot{s}}_{1}=-c_{2}{\left(n_{y0}-n_{\mathrm{yc}}\right)}^{\frac{5}{7}}+l_{1}n_{y0}+l_{2}\omega_{z0}-l_{3}d_{z0}+l_{4}\omega_{x}\omega_{y}$$

where the coefficients above are defined as follow:

$$l_{1}=c_{1}a_{34}+c_{3}\frac{{\mathrm{ga}}_{24}}{{\mathrm{va}}_{34}}$$

$$l_{2}=c_{3}a_{22}-c_{1}\frac{{\mathrm{va}}_{34}}{g}$$

$$l_{3}=-c_{3}a_{25}-c_{1}\frac{{\mathrm{va}}_{34}a_{35}}{g}$$

$$l_{4}=(J_{x}-J_{y})/J_{z}$$

According to Equation (5), the control law of SMC then can be designed.

(6) $$u_{f}=u_{\mathrm{eq}}+u_{\mathrm{vf}}$$

(7) $$u_{\mathrm{eq}}=\frac{1}{l_{3}}\left[l_{1}n_{y}+l_{2}\omega_{z}-c_{2}{\left(n_{y0}-n_{\mathrm{yc}}\right)}^{\frac{5}{7}}+l_{4}\omega_{x}\omega_{y}\right]$$

(8) $$u_{\mathrm{vf}}=\frac{1}{l_{3}}\left[\rho_{1}\tanh\left(\mu^{-1}s_{1}\right)+k_{1}s_{1}\right]$$

where $u_{\mathrm{mf}}$ is equivalent to control law, $u_{\mathrm{vf}}$ is a nonlinear control law.

### 3.2. Demonstration of Stability of Sliding Mode

The system stability is close to the performance of the controller. The nonlinear and strong coupling system of UVA studied above will be proved to satisfy Lyapunov stability. Due to the similarity among pitch, yaw and roll channels, pitch channel will be proved only here. Before, a Lemma 1 is given for the convenience of later demonstration.

Lemma 1 [34]: for an arbitrary x, when σ>0, the following non-equality exists

(9) $$\mathrm{xtanh}\frac{x}{\sigma }=\left|\mathrm{xtanh}\frac{x}{\sigma }\right|=\left|x\right|\left|\tan h\frac{x}{\sigma }\right|\geq 0$$

Demonstration:

The expression of the hyperbolic tangent function is

(10) $$\mathrm{xtanh}\frac{x}{\sigma }=x\frac{e^{\frac{x}{\sigma }}-e^{-\frac{x}{\sigma }}}{e^{\frac{x}{\sigma }}+e^{-\frac{x}{\sigma }}}=\frac{1}{e^{2\frac{x}{\sigma }}+1}x(e^{2\frac{x}{\sigma }}-1)$$

Due to

(11) $$\{\begin{array}{l} e^{2\frac{x}{\sigma }}-1\geq 0,x\geq 0\\ e^{2\frac{x}{\sigma }}-1<0,x<0 \end{array}\;$$

Then

(12) $$x(e^{2\frac{x}{\sigma }}-1)\geq 0$$

Finally

(13) $$\mathrm{xtanh}\frac{x}{\sigma }=\frac{1}{e^{2\frac{x}{\sigma }}+1}x(e^{2\frac{x}{\sigma }}-1)\geq 0$$

The demonstration of lemma 1 is finished.

To prove the stability of the designed sliding mode control system, the following Lyapunov function is established:

(14) $$v\left(x\right)=\frac{{\left[s_{1}\left(x\right)s_{1}\left(x\right)\right]}^{2}}{2}$$

The derivative of the Lyapunov function with respect to $s_{1}\left(x\right)$ can be represented as:

(15) $$\dot{v}\left(x\right)=s_{1}\left(x\right){\dot{s}}_{1}\left(x\right)$$

Substituting Equation (5) into Equation (15):

(16) $$\dot{v}\left(x\right)=s_{1}\left(x\right)\left[-c_{2}{\left(n_{y0}-n_{\mathrm{yc}}\right)}^{\frac{5}{7}}+l_{1}n_{y0}+l_{2}\omega_{z0}-l_{3}u_{f}+l_{4}\omega_{x}\omega_{y}\right]$$

Then, the derivative of the Lyapunov function can be calculated by substituting Equations (6)–(8) into Equation (16):

(17) $$\dot{v}\left(x\right)=s_{1}\left(x\right)\left[-c_{2}{\left(n_{y0}-n_{\mathrm{yc}}\right)}^{\frac{5}{7}}+l_{1}n_{y0}+l_{2}\omega_{z0}+l_{4}\omega_{x}\omega_{y}-\left(l_{1}n_{y0}+l_{2}\omega_{z0}-c_{2}{\left(n_{y0}-n_{\mathrm{yc}}\right)}^{\frac{5}{7}}\right)+l_{4}\omega_{x}\omega_{y}-\rho_{1}\tanh\left(\mu^{-1}s_{1}\right)-k_{4}s_{1}\right]$$

Equation (18) can now be simplified as

(18) $$\dot{v}\left(x\right)=-\rho_{1}s_{1}\tanh\left(\mu^{-1}s_{1}\right)-k_{1}s_{1}^{2}$$

While $\rho_{1}\;\mathrm{and}\;\mu$ are positive real numbers, according to quote 1, the following non-equality exists

(19) $$\rho_{1}s_{1}\tanh\left(\mu^{-1}s_{1}\right)\geq 0$$

Then

(20) $$-\rho_{1}s_{1}\tanh\left(\mu^{-1}s_{1}\right)\leq 0$$

$k_{1}$ is positive, then:

(21) $$-k_{1}s_{1}^{2}\leq 0$$

Ultimately, the derivative of Lyapunov function $\dot{v}$ that is equal to Equation (20) plus (21) satisfies non-equality $\dot{v}\leq 0$.

According to Lyapunov stability theory, asymptotic stability of the control system of the pitch channel has been proved. The demonstration of yaw channel and roll channel are similar to the pitch channel.

### 3.3. RBF Neural Network Algorithm

In part 3, $u_{\mathrm{vf}}$ represents the nonlinear control law.

$$u_{\mathrm{vf}}=\frac{1}{l_{3}}\left[\rho_{1}\tanh\left(\mu^{-1}s_{1}\right)+k_{1}s_{1}\right]$$

The parameter $k_{1}$ is designed using the characteristic that the RBF can accurantely approximate any continuous function accurately. At the same time, from Lyapunov stability theory, we know that if ${\;k}_{1}$ is positive, the sliding mode control satisfies Lyapunov stability.

The RBF neural network uses a three-layer forward network. The input-to-output mapping is nonlinear, while the hidden-to-output layer mapping is linear, which greatly improves the learning speed and avoids the local minimum problem. The input of the first layer is the error signal and its derivative. The second layer of the hidden layer uses the Gaussian function as the basis function; the third layer is the output layer, and the output parameter value is output. Since the three channels are independently designed, the neural network design of the parameters of the sliding mode controller of the three channels is separately designed here.

**Figure 1:**
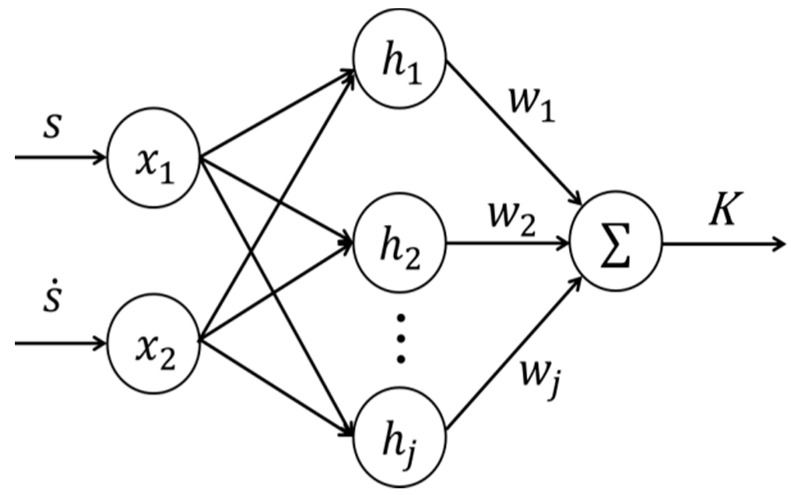
Structure of RBF neural network.

In this paper, the RBF neural network is used to adjust the gain $k_{1}$ of the nonlinear control. The neural network structure is shown in Figure 1. The neural network adopts the structure of 2-7-1.

In the RBF network structure, select $X={\left[s,\dot{s}\right]}^{T}$ as the input vector. Define the radial basis vector that makes up the neural network $H={\left[h_{1},h_{2},h_{3},h_{4},h_{5},h_{6},h_{7}\right]}^{T}$, where $h_{j}$ is Gaussian function.

$$h_{j}=\exp\left(-\frac{\left\|X-C_{j}\right\|{}^{2}}{2b_{j}^{2}}\right),j=1,2\cdots 7$$

$$C_{j}=\left[C_{1},C_{2}\right],j=1,2\cdots 7$$

where the center vector of the jth node of the network is $C_{j}$. $B={\left[b_{1},b_{2},\cdots b_{7}\right]}^{T}$ is the node center vector and output weight is $W={\left[w_{1},w_{2},\cdots w_{7}\right]}^{T}$. Therefore, the parameters’ output after the neural network calculation are:

$$k_{1}=\left|W\cdot H\right|=\left|\sum_{j=1}^{7}w_{j}\cdot h_{j}\right|$$

The learning algorithm of the base width parameter, the node center and the output weight in the above neural network can be obtained according to the gradient descent method. Let the performance indicator function be a quadratic function:

$$J=\frac{1}{2}{\left(n_{y}-n_{\mathrm{yc}}\right)}^{2}$$

Then the node center change value can be described as

$$\Delta b_{j}=-\frac{\partial J}{\partial b_{j}}=-\left(n_{y}-n_{\mathrm{yc}}\right)\cdot \frac{\partial n_{y}}{\partial b_{j}}=-\left(n_{y}-n_{\mathrm{yc}}\right)\cdot \frac{\partial n_{y}}{\partial k_{1}}\cdot \frac{\partial k_{1}}{\partial b_{j}}$$

$$\approx -\left(n_{y}-n_{\mathrm{yc}}\right)\cdot \frac{\partial k_{1}}{\partial b_{j}}\cdot \mathrm{sgn}\left(k_{1}\right)=-\left(n_{y}-n_{\mathrm{yc}}\right)\cdot w_{j}\cdot h_{j}\cdot \frac{\left\|X-C_{j}\right\|{}^{2}}{b_{j}^{3}}\cdot \mathrm{sgn}\left(k_{1}\right)$$

j=1,2⋯7

Then the node center value is:

$$b_{j}=b1_{j}+\eta \cdot \Delta b_{j}+\mu \left(b1_{j}-b2_{j}\right)$$

The base width change value is described as:

$$\Delta C_{\mathrm{ji}}=-\frac{\partial J}{\partial C_{\mathrm{ji}}}=-\left(n_{y}-n_{\mathrm{yc}}\right)\cdot \frac{\partial n_{y}}{\partial C_{\mathrm{ji}}}=-\left(n_{y}-n_{\mathrm{yc}}\right)\cdot \frac{\partial n_{y}}{\partial k_{1}}\cdot \frac{\partial k_{1}}{\partial C_{\mathrm{ji}}}$$

$$\approx -\left(n_{y}-n_{\mathrm{yc}}\right)\cdot \frac{\partial k_{1}}{\partial C_{\mathrm{ji}}}\cdot \mathrm{sgn}\left(k_{1}\right)=-\left(n_{y}-n_{\mathrm{yc}}\right)\cdot w_{j}\cdot h_{j}\cdot \frac{X-C_{\mathrm{ji}}}{b_{j}^{3}}\cdot \mathrm{sgn}\left(k_{1}\right)$$

Then the node base width is:

$$C1_{j}=C1_{\mathrm{ji}}+\eta \cdot \Delta C_{\mathrm{ji}}+\mu \left(C1_{\mathrm{ji}}-C2_{\mathrm{ji}}\right)$$

The output weight change can be described as:

$$\Delta w_{j}=-\frac{\partial J}{\partial w_{j}}=-\left(n_{y}-n_{\mathrm{yc}}\right)\cdot \frac{\partial n_{y}}{\partial w_{j}}=-\left(n_{y}-n_{\mathrm{yc}}\right)\cdot \frac{\partial n_{y}}{\partial k_{1}}\cdot \frac{\partial k_{1}}{\partial w_{j}}$$

$$\approx -\left(n_{y}-n_{\mathrm{yc}}\right)\cdot \frac{\partial k_{1}}{\partial w_{j}}\cdot \mathrm{sgn}\left(k_{1}\right)=-\left(n_{y}-n_{\mathrm{yc}}\right)\cdot h_{j}\cdot \mathrm{sgn}\left(k_{1}\right)$$

Then the output weight is:

$$w_{j}=w1_{j}+\eta \cdot \Delta w_{j}+\mu \left(w1_{j}-w2_{j}\right)$$

where η is the learning rate, μ is the learning factor, 0 < η < 1, 0 < μ < 1 is also satisfied.

## 4. Simulation Results and Discussion

In order to verify the fault-tolerant control law derived above, the numerical simulation has been performed using a mathematic model of a certain UAV. As a representative case, the nominal dynamics features are set at a speed of 0.8 Ma and altitude of 8000 m. The dynamic coefficients of a feature point of UAV are shown in Table 2:

The control parameters used for simulation are shown in the following Table 3. 

The parameters $k_{1}$, $k_{2}$ and $k_{3}$ adjusted by RBF at the above characteristic points are 2.2, 3.1 and 3.7 respectively, and all of them are greater than zero. Therefore, according to Theorem 1, the controller designed is proved to be stable.

The UAV actuator and sensor faults are simulated respectively as follows:

1. Simulation results of fault tolerance control in case of sudden failures of the actuator

**Figure 2:**
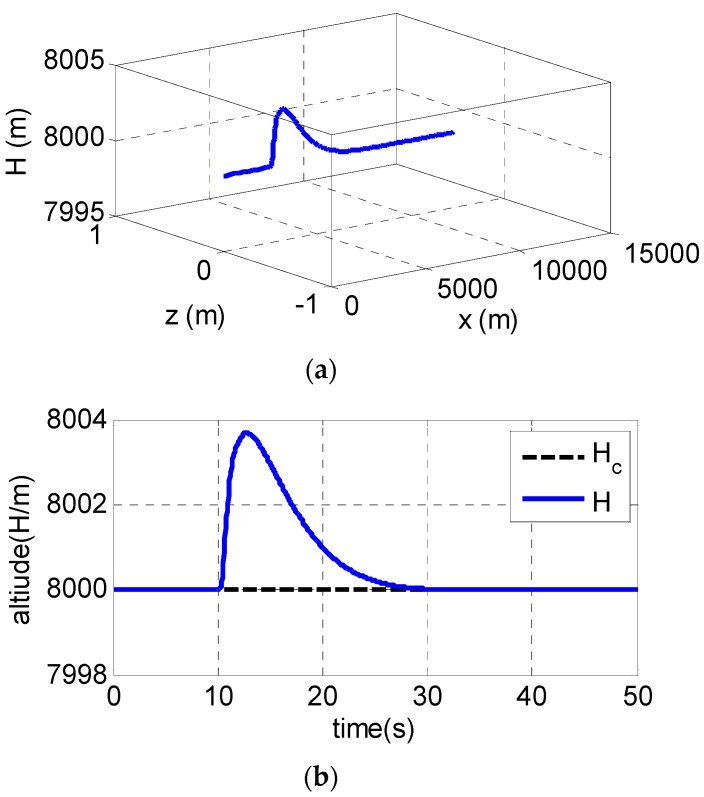

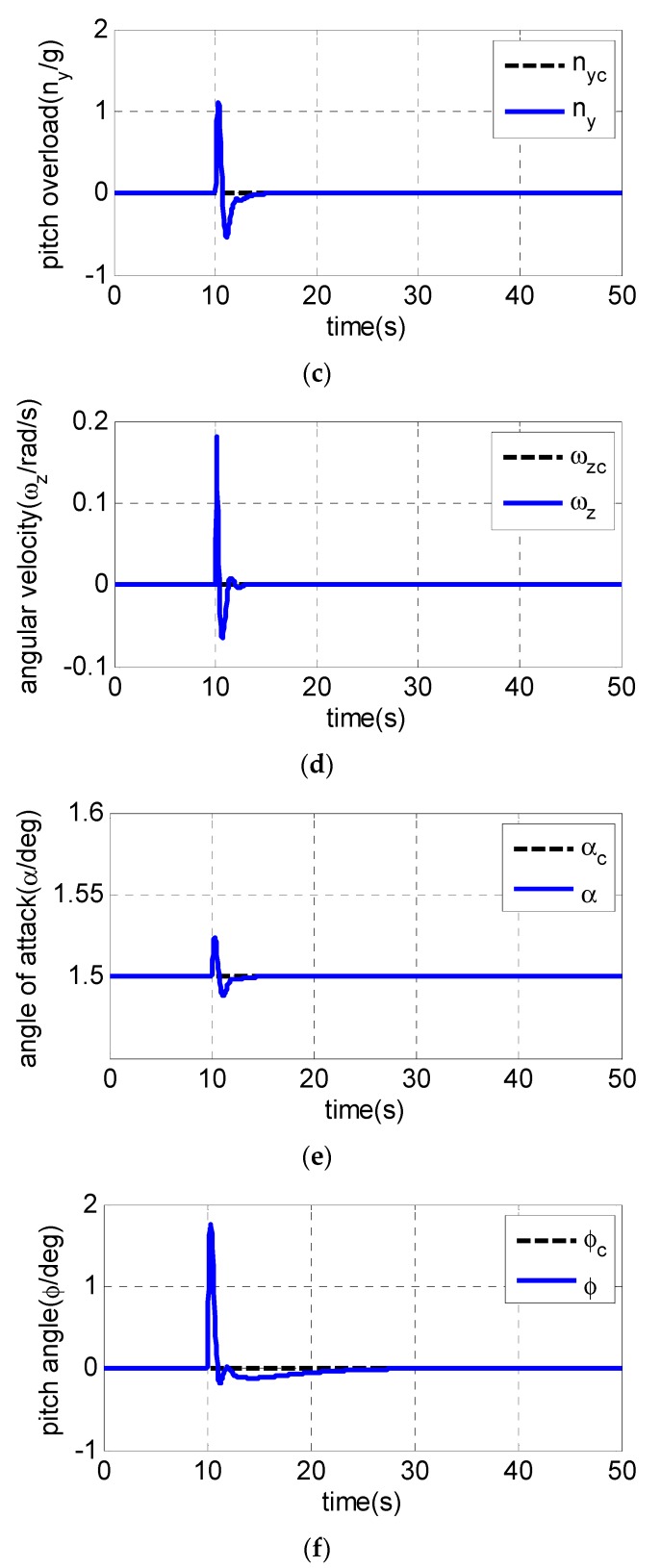
Simulation results in case of sudden failures of the actuator: (**a**) 3D trajectory; (**b**) altitude; (**c**) pitch overload; (**d**) angular velocity; (**e**) attack angle; and (**f**) pitch angle.

Pitch channel is used as an example to conduct simulations. The UAV is assumed to be cruising at a fixed altitude of 8000 m. At t = 5 s, elevator is assumed fails and the deflection of steering gear is reduced 20%. The following Figure 2 shows the simulation results:

The control of altitude uses the classical PID and the control of overload uses the fault-tolerant control designed in this paper. From the trajectory diagram in Figure 2a, during the cruise of UAV, the longitudinal trajectory has a slight fluctuation and quickly stabilizes back to the cruise trajectory when the actuator of the elevating steering gear fails; the details can be found in Figure 2b. What is more, there is no fluctuation in the trajectory of the X axis and Z axis, namely, the failure of the actuator of the pitch channel will not affect the trajectory of the yaw channel and the roll channel, indicating that the decoupling of three channels is realized by the fault-tolerant control law. 

The altitude of UAV changed slightly after the failures of the actuator, as shown in Figure 2b, and returned to the balance point of 8000 m after a period of 20 s with a maximum fluctuation of altitude of 3.8 m. As observed in Figure 2c, the pitch overload of UAV is stabilized quickly using the fault-tolerant control, namely, the UAV’s attitude is stabilized which ensures the safety of UAV while the variation of the attitude angle of UAV is shown in Figure 2d–f. Although there is a slight attitude fluctuation, it is inevitable when the fault occurs. Similarly, this situation also exists in other reports, such as Reference [35]. Compared with Reference [35], the method of the paper gives a slighter fluctuation of trajectory when the fault occurs. 

In general, it is found that the angular velocity, attitude and overload of UAV gradually reach the steady state and are also not far from steady state in the process after the actuator failure at t = 5 s using the fault-tolerant controller. The results indicate that the fault tolerant control can give a satisfied control performance and ensure the system safety of UAV even when the actuator fails.

2. Simulations results of fault tolerance control in case of sensor faults

**Figure 3:**
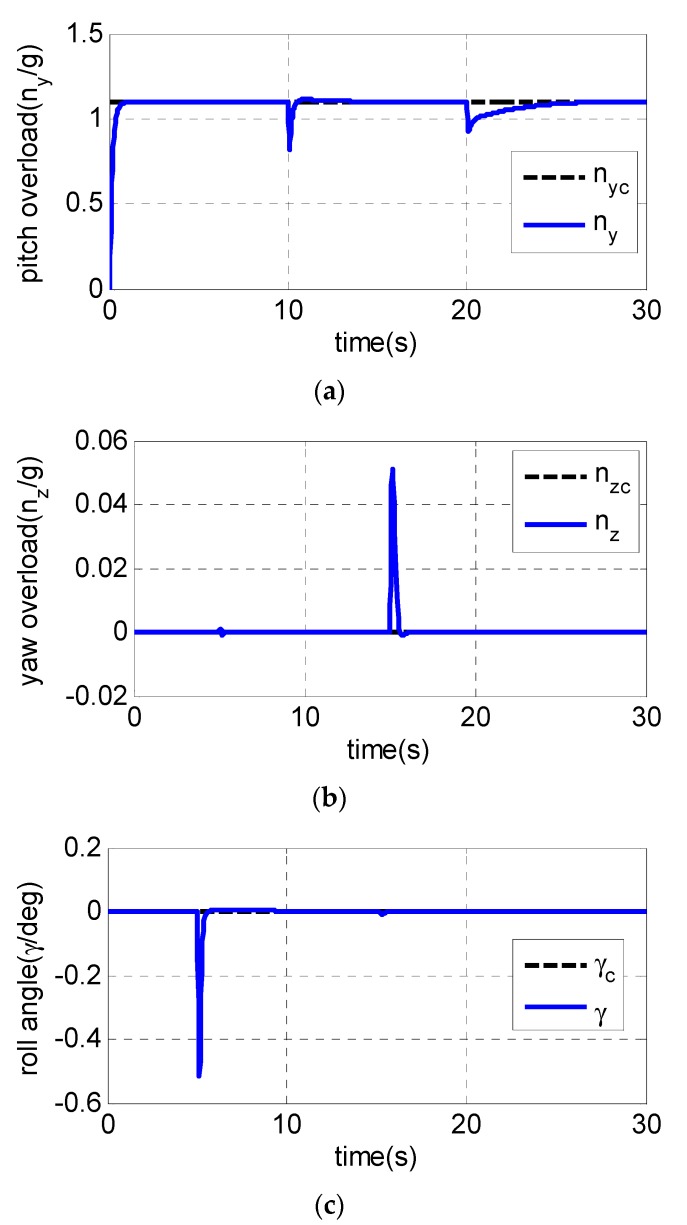
Simulation results of command tracking of three channels: (**a**) pitch overload; (**b**) yaw overload; and (**c**) roll angle.

**Figure 4:**
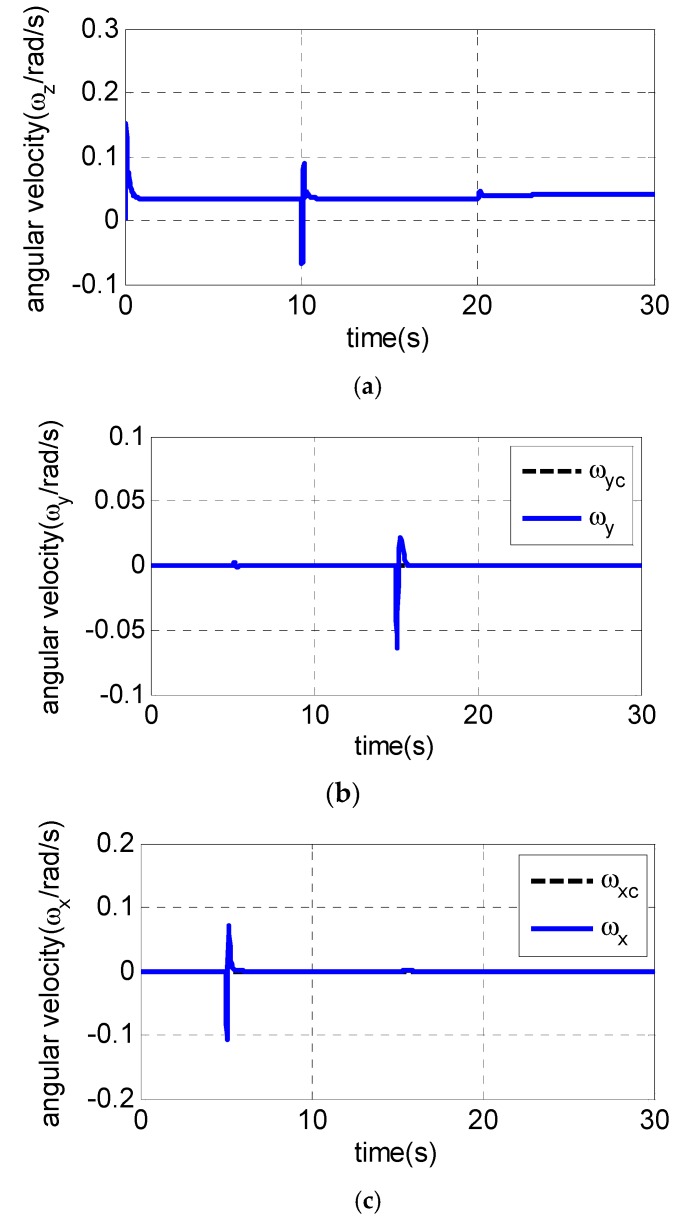
Simulation results of angular velocity of three channels: (**a**) angular velocity $\omega_{z}$ of pitch channel; (**b**) angluar velocity $\omega_{y}$ of yaw channel; and (**c**) angluar velocity $\omega_{x}$ of roll channel.

**Figure 5:**
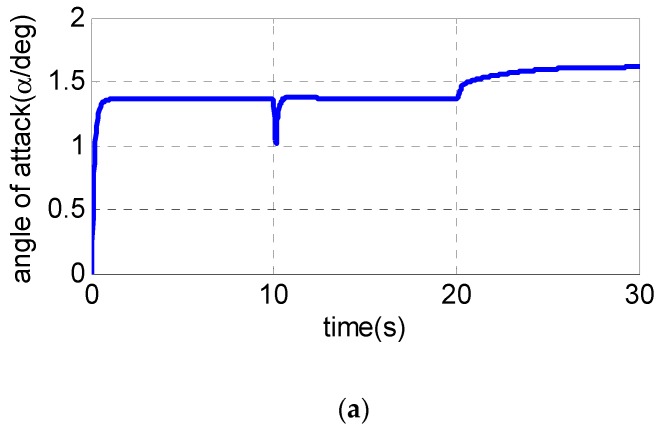

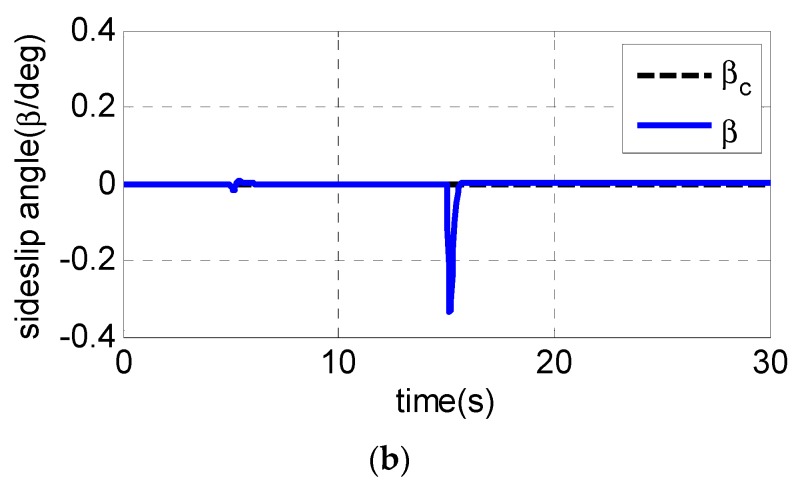
Simulation results of attack angle and sideslip angle: (**a**) attack angle and (**b**) sideslip angle.

**Figure 6:**
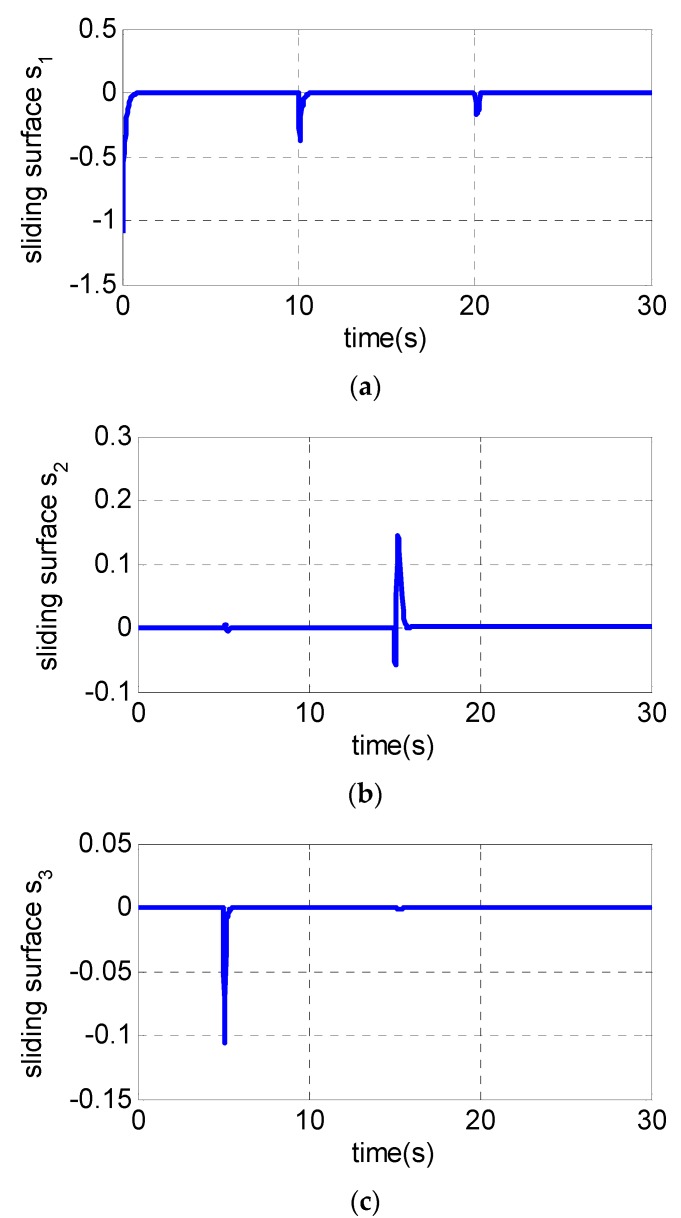
Simulation results of sliding surface of three channels: (**a**) s_1_; (**b**) s_2_ and (**c**) s_3_.

It is assumed that the UAV is climbing at an acceleration of 1.1 g in the longitudinal plane. The gyroscope of the roll channel, pitch channel and yaw channel emerge with a 30% measurement error at 5 s, 10 s and 15 s, respectively, while the pitch accelerometer fails at 20 s with the measurement error of −20%. The following Figure 3, Figure 4 and Figure 5 are the simulation results:

From Figure 3a–c, the pitch channel fails at 10 s and 20 s. Only the pitch channel is affected while there is almost no influence on the yaw channel and roll channel. The same conclusion can also be drawn to the yaw channel and roll channel, indicating that the failures of one channel sensor have little influence on the other two channels, and the decoupling of three channels is realized using the fault-tolerant control law. Figure 4a and Figure 5a show the pitch channel’s attitude. The attitude is stabilized by the pitch overload controller rapidly within 3 s after the gyroscope in the pitch channel fails at 10 s. In the case where the accelerometer fails in 20 s, the attitude returns to the steady state within 5 s. When the yaw-channel gyroscope fails at 15 s, the lateral overload fluctuates slightly and returns to the balanced state as shown in Figure 3b, Figure 4b and Figure 5b. The roll angle fluctuates with ±0.5 degree when the roll-channel gyroscope fails in 5s as shown in Figure 3c. As can be seen from Figure 6, the three channels’ sliding surfaces are smooth with slight fluctuations and rapidly recover to 0 when the sensor fails. The fault is also considered in Reference [20] but a longer time for the vehicle to regain stability is taken when a failure occurs.

In summary, Figure 3, Figure 4, Figure 5 and Figure 6 show the simulation results in the case of a sudden failure of the gyro and accelerometer. With the fault-tolerant controller, the angular velocity, attitude and overload of UAV gradually reach the steady state after sensor failures. The results indicate the fault tolerant control can give satisfied control performance of UAV even when sensor fails. It can be seen that the whole system remains steady when some sensor fails, and the fault-tolerant controller has good robustness.

## 5. Conclusions

In this paper, a sliding mode fault-tolerant controller is designed to alleviate the influence of the sensor and actuator faults of UAV. Taking the simplified model of UAV as the research object, the integrated sliding mode surface is introduced to design the terminal sliding mode control law of overload and attitude control of UAV. Then, the Lyapunov function of the system is established and its stability is proved. The sliding mode control method combined with the RBF neural network algorithm is used to design the parameters of the sliding mode controller, with which the efficiency of the design process is improved and the system chattering is minimized. The simulations of pitch, yaw and roll channels are conducted under certain sudden faults of the sensor and actuator. Results show that a good control performance of UAV is found by tracking the angular velocity, attitude angle and overload when some actuators and sensors fault, indicating that the fault-tolerant controller has good robustness which provides a guidelines for the design of FTC law of UAV with sensor and actuator Faults. Future work will focus on the controller design of the UAV’s nonlinear systems and apply it to practical engineering.

## Figures and Tables

**Table 1:** The coupling among the pitch, yaw and roll channels.

Channel	Dynamic Coupling	Pneumatic Cross Coupling	Intertie Coupling
pitch	$-\beta \cdot \omega_{x}$	—	$(J_{x}-J_{y})/J_{z}\cdot \omega_{x}\omega_{y}$
yaw	$\alpha \cdot \omega_{x}$	$b_{21}\omega_{x}-b_{28}\delta_{x}$	$(J_{z}-J_{x})/J_{y}\cdot \omega_{z}\omega_{x}$
roll	—	$-b_{14}\beta -\left(b_{12}\omega_{y}+b_{17}\delta_{y}\right)$	$(J_{y}-J_{z})/J_{x}\cdot \omega_{y}\omega_{z}$

**Table 2:** Dynamic coefficient values of UAV.

$a_{22}$	$a_{24}$	$a_{25}$	$a_{34}$	$a_{35}$	$b_{11}$	$b_{18}$
1.1532	98.3250	26.4330	0.2101	0.0302	−8.7349	0.0576
$b_{22}$	$b_{24}$	$b_{27}$	$b_{34}$	$b_{37}$	$b_{14}$	$b_{21}$
0. 2188	22.8784	35.1212	0.3107	0.1813	89.2011	0.2179

**Table 3:** The control parameters for simulation

μ	$c_{1}$	$\rho_{1}$	$c_{4}$	$\rho_{2}$	$c_{7}$	$\rho_{3}$
0.01	−1	1.2	−2.5	0.02	−4.4	0.1
